# Operationalizing the HIV prevention cascade for PWID using the integrated bio‐behavioural survey data from Ukraine

**DOI:** 10.1002/jia2.25509

**Published:** 2020-06-30

**Authors:** Kostyantyn Dumchev, Yana Sazonova, Pavlo Smyrnov, Olga Cheshun, Oksana Pashchuk, Tetiana Saliuk, Olga Varetska

**Affiliations:** ^1^ Ukrainian Institute on Public Health Policy Kyiv Ukraine; ^2^ Alliance for Public Health Kyiv Ukraine

**Keywords:** HIV prevention, prevention cascade, people who inject drugs, condoms, needle and syringe programmes, Ukraine

## Abstract

**Introduction:**

People who inject drugs (PWID) remain at high risk of HIV in many countries. The HIV prevention cascades have been proposed to replicate the success of the treatment cascades and reinvigorate the prevention programmes through improved monitoring, planning and delivery. We adapted the cascade framework to the PWID context in Ukraine, assessed gaps and analysed factors associated with achieving “access” and “effective use” outcomes.

**Methods:**

Self‐reported data on the use of prevention services and risk behaviours from the 2017 integrated bio‐behavioural survey among PWID in Ukraine were used to construct cascades for needle/syringe and condom programmes (NSP and CP). Socio‐demographic and behavioural variables were evaluated as potential correlates of cascade outcomes.

**Results:**

The NSP cascade analysis included 7815 HIV‐negative PWID. Motivation to use clean syringes was not assessed and assumed at 100%. Access to clean syringes through NSP in the past 12 months was reported by 2789 participants (35.7%). Effective use of syringes (no sharing in the past 30 days) was reported by 7405 participants (94.8%). NSP access was higher among women, individuals older than 44, and mixed drug users; while effective use was reported more frequently by men and opioid users, with no difference by age. The CP cascade analysis included 6606 (85%) of the HIV‐negative PWID who had sex in the past three months. Of those, 2282 (34.5%) received condoms, and 1708 (25.9%) reported consistent use with all partners in the past three months. Older PWID and mixed‐drug users accessed condoms more frequently; whereas younger subgroups and opioid users used them more consistently.

**Conclusions:**

Overall, the cascade framework was useful to describe the status of HIV prevention among PWID in Ukraine and to identify areas for improvement in the programming and evaluation of HIV prevention. Access to needle/syringe and condom programmes was substantially below the recommended levels. Effective use of clean syringes was reported by a vast majority of PWID, although likely affected by self‐report bias; whereas consistent condom use was infrequent. Socio‐demographic and behavioural variables showed significant associations in NSP and CP cascade analyses, with little consistency between the access and effective use outcomes.

## 1 INTRODUCTION

The number of new HIV infections is declining globally, although at a pace insufficient to reach the ambitious targets set by UNAIDS for 2020. Contrary to the global trend, some countries demonstrate an alarming growth of new infections. In Eastern Europe and Central Asia (EECA), the incidence has doubled since 2010 [1]. In EECA, and many other countries across the globe, new HIV cases remain highly concentrated among people who inject drugs (PWID) and their sexual partners [1, 2, 3].

Numerous interventions have been developed and proved to be efficacious in blocking all possible routes of HIV transmission in different populations [4]. However, their coverage, and thus the population‐level effect, remain insufficient to achieve the global targets, prompting UNAIDS to announce a “prevention crisis.”

In contrast, the progress in HIV treatment domain has been more pronounced. To a significant extent, it was achieved due to consolidated advocacy efforts instigated by the 90‐90‐90 target framework. In this framework, the key indicators are organized along the HIV treatment cascade, creating a simple and useful visualization of achievements and gaps at the main stages of HIV care [5]. This methodology was eagerly adopted by programme planners, advocacy groups, researchers and global policy makers [6].

The HIV prevention cascades have been proposed to replicate the success of the treatment cascades and reinvigorate the prevention programmes through improved monitoring, planning and delivery [7, 8]. The original concept proposed by Hargreaves and colleagues [9] was primarily informed by theory and data from the programmes addressing sexual transmission, such as condom distribution [10]. Considering other prevention methods, the authors acknowledged that some elements of the cascade may become irrelevant (e.g. adherence in voluntary medical male circumcision programmes [VMMC]) and advised to not “oversimplify HIV prevention” [8, 9]. The diversity of target populations and corresponding prevention modalities was highlighted by some critical reviews [11], indicating that the multifaceted nature of HIV prevention is not fully compatible with the linear logic of the cascade approach.

Despite the apparent need to bolster HIV prevention, there is a notable scarcity of published literature on HIV prevention cascades. Aside from early publications on condom distribution, VMMC [10], and pre‐exposure prophylaxis [12], there are a few conference abstracts [13] presenting cascades with actual program data. To the best of our knowledge, no previous publication has assessed an HIV prevention cascade for PWID, either theoretically or using real‐world data.

Ukraine has the second largest HIV epidemic in Europe, which contributed about 11% of 141,553 newly diagnosed cases in the WHO European Region in 2018 [2]. The epidemic was initially driven by PWID, who continue to have the highest prevalence among all key populations (22.6% in 2017) and to play a key role in ongoing HIV transmission [14, 15, 16]. The prevention programme, supported by international donors, expanded rapidly to reach 226,469 individual PWID with the minimum prevention package in 2017 [17]. The package is based on WHO recommendations [18] and includes provision of syringes (typically limited to 10 per day), condoms (3 per day) and peer or social worker counselling. The quality of the Ukrainian prevention programme has earned positive reviews and has been recognized as best practice in Europe by WHO [19].

In this study, we address this gap and adopt the HIV prevention cascade framework to the context of PWID programming. Using integrated bio‐behavioural survey data from Ukraine, we have constructed cascades and analysed factors associated with achieving or not achieving each stage of the continuum for two prevention interventions – needle and syringe programmes and condom distribution.

## 2 METHODS

### 2.1 Study design

For this study, we used data from the integrated bio‐behavioural survey (IBBS) among PWID conducted in November–December of 2017. Details on IBBS methodology in Ukraine are available elsewhere [16, 20]. In brief, the cross‐sectional survey was conducted in 30 cities (all 24 regional centres and six larger cities of regional significance) using respondent‐driven sampling. Eligibility criteria included presence of injection marks (verified by study personnel), self‐reported injection drug use in the past 30 days, and self‐reported age of 14 years or older. All participants completed an interviewer‐administered questionnaire and provided blood samples for the HIV test and other assessments.

### 2.2 Cascade formulation

We constructed two separate cascades for the core components of HIV prevention among PWID: needle/ syringe programmes (NSP) and condom programmes (CP).

According to the original framework [8, 9, 10], the first element of the prevention cascade is the *population at risk of HIV* and in need of the intervention. Accordingly, the NSP cascade consisted of HIV‐negative individuals who inject drugs at least once in 30 days. Considering the limitations of self‐reporting (see Discussion), we did not use risky injection practices (e.g. syringe sharing) as a criterion. The CP cascade was restricted to those PWID who are sexually active, that is, had at least one sexual partner in the past three months.

The second element of the cascade is generally conceptualized as *awareness* of HIV risk and *willingness (motivation)* to use prevention tools such as syringes and condoms. The IBBS in Ukraine did not assess the perception of personal HIV risk, nor did it assess the motivation to use the prevention tools; hence, we could not analyse this indicator. However, to retain this important step in the cascade, we assumed the motivation to use clean needles to be 100% as an injection with a new needle is less traumatic and in most cases PWID would prefer a new one if they have it available. In contrast, willingness to use condoms is not universal [21] and we, therefore, conservatively assumed the motivation to be equal to the next indicator.

In the next stage of the cascade, characterizing *access* to prevention, we included PWID who received a syringe or a condom free of charge in a prevention programme in the past 12 months. It is important to note that in Ukraine syringes are openly available for purchase in pharmacies, which is the main source of clean syringes for PWID since, in most cases, the prevention programmes cannot provide enough for each injection. Similarly, PWID can and do obtain condoms outside of the prevention programmes. The IBBS questionnaire did not include questions about access to prevention tools elsewhere, therefore our cascade indicators were limited to the access to services in the prevention programmes.

The final stage of the cascade reflects the *effective use* of the services, such as safe injection practice or protected sex. For the NSP cascade, we defined it as using only clean syringes in the past 30 days. For the CP cascade, the final indicator included PWID who always used condoms with all types of partners in the past three months.

### 2.3 Statistical analysis

#### 2.3.1 Outcome variables

All analyses were conducted in a subsample of IBBS participants who tested negative in the rapid HIV test, conducted in accordance with the WHO testing guidelines for HIV diagnosis in high prevalence settings. Access to clean syringes or condoms in prevention programmes was based on responses to the following questions: “Have you received a syringe free of charge in the past 12 months?” and “Have you received condoms free of charge in the past 12 months?”. Effective use of syringes was determined by one question: “During the past 30 days, have you injected drugs with a syringe previously used by another person?”. Consistent use of condoms was determined if the participant answered “Always” to the question, “How frequently did you use condom with this partner in the past three months?” for each of the four types of partners – regular, casual and commercial, as a client or a sex worker.

#### 2.3.2 Predictors

We used key socio‐demographic and behavioural variables as potential predictors for the cascade outcomes, including: age, sex, education, marital status, monthly income, duration of injection drug use and drug type that was injected during the past 30 days. The drug type was categorized as: exclusive opioid use – heroin, opium, desomorphine, home‐made opioids, illegal methadone or buprenorphine; exclusive stimulant use – amphetamines, methamphetamines, cocaine, synthetic cathinones (“bath salts”); mixed use of opioids and stimulants in any combination during the same time period or other drug use.

In the bivariate analysis, the association was tested using chi‐square test. Variables significant at *p* < 0.1 level were included in the multivariable logistic regression analysis. The final model with the best fit was selected via a backward stepwise technique using Wald test. Explanatory variables were removed one at a time if they were not associated with an outcome at 5% level of significance. Age and sex variables were retained in all models, even when they did not have a significant association with the outcomes.

Data were analysed using SPSS v.23 (IBM Corporation, Armonk, NY, USA).

### 2.4 Ethical approval

All procedures in studies involving human participants were performed in accordance with the ethical standards of the Institutional Research Committee and with the 1964 Helsinki declaration and its later amendments or comparable ethical standards.

Prior to enrolment into the study, all participants were provided with comprehensive information about the study and signed a consent form. The study was approved by the Institutional Review Board of the Ukrainian Institute on Public Health Policy (Kyiv, Ukraine) and was reviewed for human subject issues in the research determination process by the Centers for Disease Control and Prevention (Atlanta, GA, USA).

## 3 RESULTS

Of the total 10,076 PWID recruited in the IBBS, 2,261 tested HIV positive and 7,815 tested HIV negative, resulting in 22.4% HIV prevalence. The median age of participants was 35 years (IQR 30 to 40) and 18% of the sample were women.

**Figure 1:**
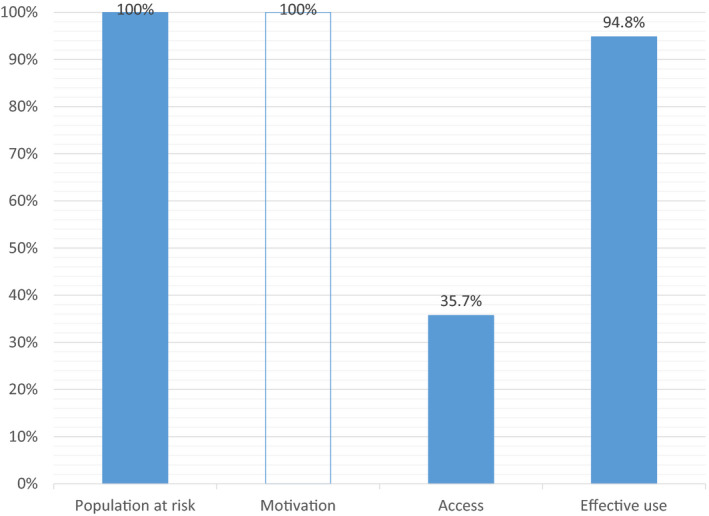
Needle/syringe programs cascade for HIV negative people who inject drugs in Ukraine. Population at risk is defined as people injecting drugs at least once in the past 30 days. Motivation is not measured and assumed at 100%. Access to service is defined as receiving syringes from prevention programs in the past 12 months. Effective use is defined as using only clean syringes in the past 30 days.

Figure 1 shows the needle/syringe programmes (NSP) cascade and Table 1 provides disaggregation by key socio‐demographic and drug use strata. Receiving clean syringes from the prevention programmes at least once in the past 12 months was reported by 2789 of participants (35.7%), whereas the exclusive use of clean syringes in the past 30 days was reported by 7405 (94.8%). Both behaviours were reported by 2685 participants (36.2% of those using only clean syringes or 96.2% of those receiving the service). Among the subcategories, NSP access was significantly higher among women, older individuals, those with longer injection careers, those with lower income, and opioid or mixed drug users (as compared to exclusive stimulant users). There was no statistical difference by family status and education. All variables in the multivariable model, except monthly income, demonstrated an independent and significant effect. Effective use of prevention was more prevalent among men, those with higher education, lower income, and opioid users. There was no difference by age, injection duration, and family status. The multivariable models largely confirmed the results of the univariate analysis, except for the effect of income.

**Table 1:** Needle/syringe programmes cascade and associated factors for HIV‐negative people who inject drugs in Ukraine

	Population at Risk^a^	Access to service^b^						Effective use^c^					
					Multivariable model						Multivariable model		
	N	n	%	χ^2^ *p*‐value	AOR	95% CI	*p*‐value	n	%	χ^2^ *p*‐value	AOR	95% CI	*p*‐value
Total	7815	2789	35.7%					7405	94.8%				
Sex													
Male	6588	2310	35.1%	0.007	ref.			6278	95.3%	<0.001	ref.		
Female	1225	479	39.1%		1.4	1.2 to 1.6	<0.001	1127	92.0%		0.6	0.4 to 0.7	<0.001
Age													
≤24	656	130	19.8%	<0.001	ref.			619	94.4%	0.888	ref.		
25 to 44	6290	2292	36.4%		1.6	1.3 to 2.0	<0.001	5963	94.8%		0.9	0.7 to 1.3	0.719
≥45	869	367	42.2%		2.0	1.5 to 2.5	<0.001	823	94.7%		0.8	0.5 to 1.3	0.475
Injection duration													
≤2 years	716	127	17.7%	<0.001	ref.			676	94.4%	0.659			
>2 years	7078	2656	37.5%		2.3	1.8 to 2.8	<0.001	6711	94.8%				
Family status													
Live alone	3279	1164	35.5%	0.774				3111	94.9%	0.757			
Live with partner	4534	1625	35.8%					4294	94.7%				
Education													
<High school	1264	444	35.1%	0.843				1177	93.1%	0.01	0.6	0.4 to 0.8	0.002
High school	4893	1746	35.7%					4646	95.0%		0.8	0.6 to 1.1	0.222
>High school	1656	599	36.2%					1582	95.5%		ref.		
Monthly income													
<120 USD	3222	1228	38.1%	<0.001				3029	94.0%	0.03			
120 to 400 USD	3997	1381	34.6%					3815	95.4%				
401 to 800 USD	493	147	29.8%					468	94.9%				
>800 USD	101	33	32.7%					93	92.1%				
Type of drugs injected in 30 days													
Only opioids^d^	4788	1770	37.0%	<0.001	2.2	1.8 to 2.6	<0.001	4592	95.9%	<0.001	0.7	0.5 to 1.0	0.051
Only stimulants^e^	1053	232	22.0%		ref.			991	94.1%		ref.		
Mix or other^f^	1974	787	39.9%		1.8	1.6 to 2.2	<0.001	1822	92.3%		1.4	1.1 to 2.0	0.015

AOR, adjusted odds ratio; CI, confidence interval; USD: United States Dollars.

a People injecting drugs at least once in the past 30 days;

b receiving syringes from prevention programmes in the past 12 months;

c using only clean syringes in the past 30 days;

d exclusive use of heroin, opium, desomorphine, home‐made opioids, illegal methadone or buprenorphine;

e exclusive use of amphetamines, methamphetamines, cocaine, synthetic cathinones (“bath salts”);

f use of opioids and stimulants in any combination during the same time period or other drug use.

**Figure 2:**
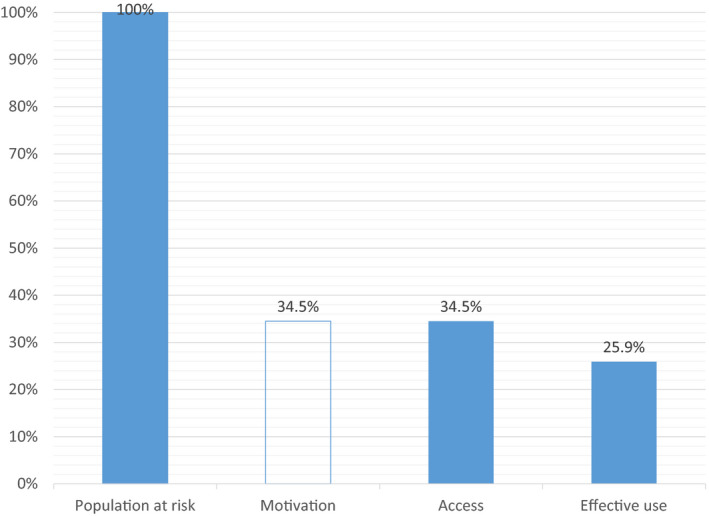
Condom programs cascade for HIV negative people who inject drugs in Ukraine. Population at risk is defined as people injecting drugs at least once in the past 30 days and having sex in the past 3 months. Motivation is not measured and assumed equal to the next indicator. Access to service is defined as receiving condoms from prevention programs in the past 12 months. Effective use is defined as always using condom with all partners in the past 3 months.

The condom programmes (CP) cascade is shown in Figure 2 and Table 2. The risk of sexual transmission, defined in this analysis as having at least one sexual partner in the past three months, was reported by 6606 (85%) of all HIV‐negative PWID. Access to free condoms in the prevention programmes in the past 12 months was reported by 2282 (34.5%) of those at risk, which is only marginally lower than what was reported for NSP. Effective use of condoms, in contrast to syringes, was reported by only 1708 (25.9%) participants. Combination of access to services and effective use was reported by 673 participants, meaning that only 39.3% of all at‐risk participants and 29.5% of those receiving the service report always using condoms. Receipt of condoms in prevention programmes in the past 12 months was higher among older individuals, those who have longer injection history, have lower income and are mixed drug users. These associations were confirmed in the multivariable model, where the effect of gender also became significant (aOR = 1.2 [95% CI 1.0 to 1.4] for women compared to men). Consistent condom use was higher among men, younger PWID, those with shorter injection history, living alone, having a lower income and among opioid users. The multivariable models confirmed the associations of consistent condom use with gender, age and family status.

**Table 2:** Condom programmes cascade for HIV‐negative people who inject drugs in Ukraine

	Population at risk^a^	Access to service^b^						Effective use^c^					
					Multivariable model						Multivariable model		
	N	n	%	χ^2^ *p*‐value	AOR	95% CI	*p*‐value	n	%	χ^2^ *p*‐value	AOR	95% CI	*p*‐value
Total	6606	2282	34.5%					1708	25.9%				
Sex													
Male	5543	1902	34.3%	0.379	ref.			1524	27.5%	<0.001	ref.		
Female	1063	380	35.7%		1.2	1.0 to 1.4	0.012	184	17.3%		0.7	0.5 to 0.8	<0.001
Age													
<25	574	130	22.6%	<0.001	ref.			188	32.8%	<0.001	ref.		
25 to 44	5427	1907	35.1%		1.3	1.1 to 1.7	0.009	1395	25.7%		0.7	0.5 to 0.8	<0.001
≥45	605	245	40.5%		1.6	1.2 to 2.1	0.001	125	20.7%		0.5	0.4 to 0.6	<0.001
Injection duration													
≤2 years	623	116	18.6%	<0.001	ref.			187	30.0%	0.014			
>2 years	5967	2162	36.2%		2.2	1.7 to 2.7	<0.001	1519	25.5%				
Family status													
Live alone	2241	771	34.4%	0.87				907	40.5%	<0.001	ref.		
Live with partner	4365	1511	34.6%					801	18.4%		0.3	0.3 to 0.4	<0.001
Education													
<High school	1014	339	33.4%	0.65				253	25.0%	0.182			
High school	4156	1437	34.6%					1057	25.4%				
>high school	1436	506	35.2%					398	27.7%				
Monthly income													
<120 USD	2528	933	36.9%	0.002				661	26.1%	0.01			
120 to 400 USD	3538	1192	33.7%					940	26.6%				
401 to 800 USD	446	129	28.9%					88	19.7%				
>800 USD	94	28	29.8%					19	20.2%				
Type of drugs injected in 30 days													
Only opioids^d^	3964	1407	35.5%	<0.001	1.9	1.6 to 2.3	<0.001	1071	27.0%	0.03			
Only stimulants^e^	920	213	23.2%		ref.			220	23.9%				
Mix or other^f^	1722	662	38.4%		1.7	1.4 to 2.0	<0.001	417	24.2%				

AOR, adjusted odds ratio; CI, confidence interval.

a People injecting drugs at least once in the past 30 days and had sex in the past three months;

b receiving condoms from prevention programmes in the past 12 months;

c always using condom with all partners in the past three months;

d exclusive use of heroin, opium, desomorphine, home‐made opioids, illegal methadone or buprenorphine;

e exclusive use of amphetamines, methamphetamines, cocaine, synthetic cathinones (“bath salts”);

f use of opioids and stimulants in any combination during the same time period or other drug use.

## 4 DISCUSSION

### 4.1 Cascade status and implications for programming

In this analysis, we applied the HIV prevention cascade framework to the data from the 2017 IBBS among PWID in Ukraine. Overall, the analysis was helpful to better understand the current status of HIV prevention among PWID in Ukraine. The cascade demonstrated that access to prevention interventions remains suboptimal, with just over one third of HIV‐negative PWID accessing NSP to receive syringes in the past 12 months. The data also showed that the majority of PWID obtain clean injection equipment elsewhere, leading to the low reported level of syringe sharing. This last indicator has to be interpreted with caution, considering the self‐reporting bias (see below).

Access to CP was similar to that of NSP, explained by the fact that these services are usually co‐provided. However, in contrast to clean syringes, consistent use of condoms was reported by only a quarter of sexually active PWID. Similar to clean syringes, the majority of condom users were not covered by CP and purchased condoms at their own expense.

It should be noted that the service access indicators were based on the least stringent definition of receiving service at least once in the past 12 months. Applying more rigorous criteria, such as frequency or regularity, would decrease the estimates substantially.

The subgroup analysis revealed that uptake of NSP was higher among women, older and more experienced injectors, as well as users of opioids or a combination of drugs. However, the effective syringe use was slightly less frequent among women and mixed drug users. CP was more frequently accessed by older participants with longer injection history, and by opioid and mixed drug users. This also did not translate into higher rate of effective use, which was notably higher among males, young people and those living alone. Overall, the associations we found between the age and drug type with programme access and risk behaviour is consistent with other evidence indicating that PWID with riskier behaviour are seeking prevention services more intensively than those with lower risk [22, 23].

Our findings confirm that HIV prevention efforts among PWID should be intensified through increase in both NSP and CP, particularly among specific subgroups such as young people and stimulant users. Unlike with treatment, which is indicated for all HIV‐infected, universal coverage by most prevention interventions is not realistically attainable and may also not be necessary to achieve the reduction of incidence on the population level. The WHO tool [18] recommends a 60% NSP coverage target with at least 200 syringes per person per year, albeit recognizing that this number may not be sufficient to provide clean syringes for each injection.

### 4.2 Framework adaptation and recommendations for evaluation

While the proposed cascade framework should be applicable to all key populations and prevention methods [8], we faced substantial challenges in adapting it to the PWID context. At the first step of the NSP cascade, the population in need of prevention services could be defined in several ways. We used the current definition of the PWID key population in Ukraine, which entails using drugs by injection at least once in the past month, regardless of specific behaviours directly associated with HIV transmission risk (e.g. syringe sharing). It can be speculated that other people who inject less frequently or are at risk of relapsing to injection (non‐injection drug users, OAT patients) may also benefit from prevention services. Determination of sexual risk is no less complex, as it is affected not only by frequency of activity, but also by types of partners, specific practices, and use of protection. Similarly to non‐injectors, people who have not had sex in the recent past may also re‐engage in sexual activity and thus should not be excluded from condom provision.

Moreover the framework assumes that only HIV‐negative population should be included in the cascade. In reality, the prevention programmes for PWID never distinguish clients based on HIV status and equally serve HIV positive, HIV negative, and people with unknown HIV status because risk reduction of HIV acquisition is as important as reduction of transmissibility among people living with HIV. From this standpoint, the inclusion of HIV‐positive PWID in the cascade and analysis of HIV status as one of the factors influencing access to and effective use of services may be justifiable.

For the next step of the cascade, we assumed the motivation to use clean needles to be 100% because injecting with a new needle is less traumatic. Some studies have found that PWID may intentionally share needles in some circumstances [24, 25]. However, reports of such practices became less frequent in the era of the grown HIV epidemic and nearly universal knowledge of HIV risk among PWID [21]. Motivation to use condoms, in contrast, is determined by other factors and is far from universal, leading to an assumption that only those who received the service were motivated.

The service access indicator appeared to be the most challenging to operationalize. Unlike treatment, prevention tools are available outside of prevention programmes and are widely used for purposes other than HIV prevention. The IBBS in Ukraine did not measure access to prevention tools elsewhere; therefore, our cascade indicator was limited to receipt of the tools from the programmes. We believe that this approach is adequate to serve programming purposes, such as assessment and planning of coverage overall and in specific subgroups. It is also important to understand that, compared to individual purchase of prevention tools at a pharmacy, services provided by HIV prevention programmes are more complex and serve multiple synergistic purposes.

We defined the final element of the cascade, the effective use of prevention services, as consistent use of the condoms or the clean syringes. Unlike in the treatment cascade, where viral suppression is almost exclusively a result of treatment, behaviours that prevent HIV are often practiced without accessing specific programmes. As shown in our data, nearly two thirds of PWID reporting consistent use of prevention tools did not receive them through the prevention programmes. Such disconnect between the two indicators may seem to contradict the underlying sequential cascade logic. In the prevention context, however, it makes sense because safer behaviours are facilitated not only by direct provision of prevention services but also by educating about harm reduction approaches and motivating to obtain tools from other sources.

Several amendments to the IBBS instruments can be made in order to enable a more accurate and complete estimation of the HIV prevention cascade. To assess motivation, the second element of the cascade, IBBS should include questions to measure the perceived HIV risk and motivation to use prevention services. Additional questions are needed to comprehensively measure access to prevention tools within and outside of prevention programmes. It is also important to consider the temporal dimension and assess the frequency and regularity of tools uptake. Lastly, questions related to all cascade indicators should use the same time frame (i.e. past twelve or three or one month).

### 4.3 Limitations

Several limitations should be considered in interpreting our findings. First, the IBBS data on service uptake are vulnerable to reporting bias. When responding to the questions, participants may not be fully aware that syringes acquired through pharmacy exchange sites or through secondary exchange volunteers may come from the prevention programmes. This could partially explain the notable discrepancy with the programmatic data indicating higher levels of access [17]. The risk behaviour indicators, especially the most straightforward ones regarding syringe sharing, are likely affected by the social desirability bias due to the ubiquitous exposure of Ukrainian PWIDs to HIV‐related information and regular surveys. This could lead to a substantial overestimation of the effective use of services element.

As described in Methods, several limitations in the IBBS data source led to compromises in adapting the cascade methodology. These included the absence of motivation and risk perception measures, lack of data on the uptake of prevention tools outside of the programmes, and different time frames in the access and effective use‐related questions.

Our analyses did not include other types of risk behaviours, such as back‐ and front‐loading, using pre‐filled syringes, or container sharing, because these practices are not directly affected by the availability of clean syringes. Substantial prevalence of these behaviours may contribute to ongoing HIV transmission among PWID despite active NSP in Ukraine. If the “effective use” definition would account for these practices, the estimates of the last stage of the cascade would decrease significantly.

## 5 CONCLUSIONS

The generic HIV prevention cascade framework was proposed some time ago to reinvigorate the HIV prevention programme [7, 8]. The first real‐life cascade analyses used data from condom distribution and pre‐exposure prophylaxis programmes [10, 12], and this study is the first such example for PWID. We estimated the NSP and CP cascades for PWID in Ukraine using the IBBS survey data and analysed the programming gaps, as well as demographic and behavioural factors associated with achieving the cascade outcomes. Access to NSP and CP was substantially below the recommended level, especially among men and younger PWID. In contrast, effective use of clean syringes was reported by the vast majority of PWID, likely affected by self‐report bias. Consistent use of condoms was infrequent.

The analysis also revealed conceptual challenges in applying the cascade framework to the context of HIV prevention among PWID, primarily caused by complex, non‐linear causal pathways between the prevention interventions and desired outcomes. Overall, the cascade framework was useful to describe the status of HIV prevention among PWID in Ukraine and to identify areas for improvement in programming as well as evaluation of HIV prevention.

## COMPETING INTERESTS

The authors declare that they have no competing interests.

## AUTHORS' CONTRIBUTIONS

KD, YS, OV, PS and TS conceptualized the analysis approach. KD and YS analysed the data. YS designed and managed all stages of IBBS survey. OC and OP managed program data, contributed to program data analysis. TS contributed to IBBS survey design. KD and YS wrote the paper. All authors have read and approved the final manuscript.
